# Supplementary material for: Unveiling the full picture of malnutrition in Sub-Saharan Africa: The extended composite index of anthropometric failure among children under-5 in the SDG era
Source: PLoS One. 2025 Oct 3;20(10):e0333785. doi: 10.1371/journal.pone.0333785 (PMC12494244; doi:10.1371/journal.pone.0333785)
Supplement: S1 Table — (DOCX) [file pone.0333785.s001.docx]

**Table S1: List of countries and survey years used for the current study titled “Unveiling the Full Picture of Malnutrition in Sub-Saharan Africa: The Extended Composite Index of Anthropometric Failure among Children Under-5 in the SDG Era”.**

| S.N | Countries | Survey Year | African Region |
| --- | --- | --- | --- |
| 1 | Kenya | 2022 | Eastern Africa |
| 2 | Ethiopia | 2019 |  |
| 3 | Madagascar | 2021 |  |
| 4 | Rwanda | 2019 |  |
| 5 | Tanzania | 2022 |  |
| 6 | Uganda | 2016 |  |
| 7 | Mozambique | 2022 |  |
| 8 | Benin | 2017 | Western Africa |
| 9 | Burkina Faso | 2021 |  |
| 10 | Côte d'Ivoire | 2021 |  |
| 11 | Gambia | 2020 |  |
| 12 | Ghana | 2022 |  |
| 13 | Guinea | 2018 |  |
| 14 | Liberia | 2019 |  |
| 15 | Mali | 2018 |  |
| 16 | Nigeria | 2018 |  |
| 17 | Senegal | 2023 |  |
| 18 | Sierra Leone | 2019 |  |
| 19 | Burundi | 2017 | Central Africa |
| 20 | Cameroon | 2018 |  |
| 21 | Gabon | 2019 |  |
| 22 | Mauritania | 2021 | Northern Africa |
| 23 | Angola | 2016 | Southern Africa |
| 24 | Malawi | 2016 |  |
| 25 | South Africa | 2016 |  |
| 26 | Zambia | 2018 |  |
