# Supplementary material for: Unveiling the full picture of malnutrition in Sub-Saharan Africa: The extended composite index of anthropometric failure among children under-5 in the SDG era
Source: PLoS One. 2025 Oct 3;20(10):e0333785. doi: 10.1371/journal.pone.0333785 (PMC12494244; doi:10.1371/journal.pone.0333785)
Supplement: Attachment — Submitted filename: Reviewer Report.docx [file pone.0333785.s002.docx]

**Title of Manuscript**: *Unveiling the full picture of malnutrition: Extended composite index of anthropometric failure among children under-5 in Sub-Saharan Africa: In the SDG era*

This manuscript presents a comprehensive analysis of child malnutrition in Sub-Saharan Africa, focusing on the Extended Composite Index of Anthropometric Failure (ECIAF). The study explores the prevalence and regional disparities of ECIAF among children under five years old, offering insights into the multifaceted nature of malnutrition. By examining key determinants at individual, maternal, household, and community levels, the authors provide a nuanced understanding of the factors contributing to childhood malnutrition in the context of the Sustainable Development Goals (SDGs). This research is timely and relevant, addressing a pressing public health issue and proposing actionable policy recommendations. However, I have some comments to improve the manuscript as follows:

**Introduction Section:**

- The introduction clearly contextualizes the significance of the study and the relevance of ECIAF in addressing child malnutrition. However, there is room for improvement in the transition between traditional indices and ECIAF. Adding a linking sentence between these two concepts would enhance the overall flow.
- The explanation of why traditional indices fall short in capturing the multifaceted nature of malnutrition could be clarified further.
- **Line 69-71**: Clarify why traditional indices fall short in capturing the full scope of malnutrition. Consider revising: "Traditional indices such as weight-for-age, height-for-age, and weight-for-height have been instrumental in monitoring child nutrition, but they often fail to capture the multifaceted nature of malnutrition."
- **Line 74-77**: Add references to support claims about long-term health outcomes related to malnutrition. Also, refine the sentence for clarity: "Malnutrition in children under five includes stunting (low height-for-age), wasting (low weight-for-height), and underweight (low weight-for-age), all of which indicate immediate nutritional deficiencies."
- **Line 101-106**: Simplify the phrasing: "absence of a standardized composite measure" rather than "lack of a standardized composite measure."
- **Line 82**: Strengthen the paragraph discussing the SDGs by linking ECIAF’s relevance directly to these goals.
- **General suggestion**: Strengthen the introduction by explicitly stating how ECIAF can contribute to achieving the SDGs and can serve as a transformative tool in addressing malnutrition in Sub-Saharan Africa.

**Methods Section:**

- The description of the two-stage stratified cluster sampling is well-done but could benefit from additional details, particularly on how strata were defined (e.g., by region, urban/rural). This would improve the understanding of the sample's representativeness.
- **Sampling Procedure**: Add more detail on how strata were defined to clarify how different regions or population groups were represented.
- **Data Collection Period**: Include the specific period during the SDG era when the data was collected. This is important to provide context for the results.

**Statistical Methods**:

- A brief explanation of why multilevel binary logistic regression was chosen would help clarify its relevance.
- Consider including **sensitivity analysis** or robustness checks to strengthen the validity of the results.

**General suggestions**:

- **ECIAF Definition**: Clarify the categories used in the ECIAF calculation (Groups A-H). Providing a clearer and more structured presentation will improve readability.
- **Statistical Model Details**: Add a brief explanation of the statistical model fitting procedure (e.g., likelihood ratio tests, AIC) to improve transparency.

**Results Section:**

- The results section does a good job presenting the ECIAF prevalence. However, phrasing can be improved for clarity. For instance, instead of “the prevalence of ECIAF was 36% (95% CI: 33%, 40%)”, it could be reworded for better readability.
- **Phrasing**: "The overall pooled prevalence of ECIAF in children under five in Sub-Saharan Africa was 36%, with a 95% confidence interval ranging from 33% to 40%."
- **Subgroup Analyses**: The descriptions of subgroup prevalence rates (e.g., for East Africa, West Africa) could be better organized. For example, when presenting the regional differences in prevalence, include clearer transitions and structure.
- **Odds Ratios**: Provide a brief interpretation of odds ratios (AORs) for non-statistical readers. Explain their practical implications for public health.

**Discussion Section:**

- Some sentences are complex and could benefit from simplification. For example, the list of factors associated with ECIAF in children under 5 years could be broken down into clearer subpoints.
- **Sentence Structure**: Break down complex sentences like "The risk of having ECIAF was positively associated with child characteristics..." into more digestible parts.
- **Policy Implications**: The discussion could more strongly emphasize the **public health implications** of these findings. Specifically, consider elaborating on how the results can inform policy changes related to maternal education, child health, and nutrition.

**Implications, Applications, Limitations, and Conclusions:**

**Implications**:

- The implications are well-articulated but can be made more concise to improve readability. For example, consider simplifying the phrasing: "Strengthening maternal health services, promoting family planning, improving socioeconomic conditions, and enhancing access to health information through media" could be shortened to "Strengthening maternal health services, improving family planning, and promoting socio-economic development."

**Applications**:

- While the applications are comprehensive, the section would benefit from linking the findings more explicitly to specific interventions. Consider adding a sentence that connects the study’s results to practical applications.
  - Suggested revision: "The findings highlight the need for targeted interventions, such as improving maternal education and increasing access to healthcare, to address ECIAF."

**Limitations**:

- The limitations section appropriately acknowledges the cross-sectional nature of the study. However, it could be strengthened by discussing potential measurement biases from secondary data sources and missing variables like media exposure and distance to healthcare facilities.
- **Future Research**: Suggest that future research could overcome these limitations by using longitudinal designs and exploring additional factors like healthcare access and social determinants of health.

**Conclusions**:

- The conclusions effectively summarize the findings, but could benefit from a stronger emphasis on **policy implications**. For example, suggest specific actions such as improving maternal education and providing targeted nutrition programs for vulnerable populations.
