# Supplementary material for: Unveiling the full picture of malnutrition in Sub-Saharan Africa: The extended composite index of anthropometric failure among children under-5 in the SDG era
Source: PLoS One. 2025 Oct 3;20(10):e0333785. doi: 10.1371/journal.pone.0333785 (PMC12494244; doi:10.1371/journal.pone.0333785)
Supplement: Attachment — Submitted filename: Comments 13648.docx [file pone.0333785.s003.docx]

**Title and Abstract**

**Strengths:**

The title is descriptive and informative, clearly reflecting the study’s aim and population of interest.

The abstract concisely presents the background, methods, results, and conclusions.

**Areas for improvement:**

The abstract could better reflect the novel aspects of the Extended Composite Index of Anthropometric Failure (ECIAF), possibly with a brief explanation of how it surpasses traditional measures.

The use of acronyms like ECIAF could be confusing to non-technical readers in the abstract; consider spelling it out first.

**Introduction**

**Strengths:**

The introduction is well-grounded in global health goals (SDGs) and articulates the importance of accurate malnutrition assessment.

It outlines the rationale for using ECIAF and connects it to policy relevance.

**Areas for improvement:**

The literature review could be more current, with clearer referencing to similar studies post-2020.

The justification for choosing ECIAF over other composite measures could be better articulated with empirical comparisons.

**Methods**

**Strengths:**

The use of a large, representative dataset across 26 Sub-Saharan African countries is commendable.

The stratification into individual- and community-level factors and use of multilevel modeling enhance robustness.

**Areas for improvement:**

The operational definition of ECIAF, while technically accurate, is overly dense. A simplified diagram or table might help.

More detail is needed on ethical considerations, especially since secondary data use still carries responsibility for safeguarding privacy.

The variable selection process (p-value < 0.2 + literature + expert judgment) is common but should be justified more explicitly for transparency.

**Results**

**Strengths:**

Clear presentation of descriptive data, subgroup analysis, and multivariate findings.

Use of figures and tables helps to break down complex results.

**Areas for improvement:**

The writing could be more fluid; it reads as a data dump in parts. Focus on highlighting trends, not just numbers.

Regional variations are well-presented, but the manuscript does not delve deeply into why these differences exist—more interpretive insight is needed.

**Discussion**

**Strengths:**

The discussion covers multiple determinants of ECIAF and links findings to prior research.

There’s a commendable effort to draw policy implications from the data.

**Areas for improvement:**

There’s repetition from the results section; consider condensing and focusing on interpretation.

The discussion lacks a critical reflection on the study's limitations. For example, potential bias from self-reported birth sizes or unmeasured confounders should be acknowledged.

The regional comparison could benefit from linking findings to historical, cultural, or policy-based explanations.

**Conclusion**

**Strengths:**

It concisely summarizes key findings and policy relevance.

Emphasizes practical interventions such as improving maternal education and health access.

**Areas for improvement:**

A clearer statement on the novelty of ECIAF and its potential for replication across other regions would strengthen the final impact.

A recommendation for future research directions (e.g., longitudinal studies or validation of ECIAF) would add value.

**Language and Structure**

**Strengths:**

Generally readable and logically structured.

Tables and figures are appropriate and informative.

**Areas for improvement:**

Grammatical inconsistencies and typos (e.g., “unde-5” instead of “under-5,” “Sothen Africa”) should be corrected for professionalism.

Some paragraphs are too long or overly technical; consider simplifying for broader audiences.

**Overall Assessment**

This manuscript tackles an important public health issue with methodological rigor and a large dataset. The ECIAF provides an innovative approach to understanding malnutrition beyond standard indices. However, the manuscript would benefit from clearer articulation of novelty, improved interpretation of regional differences, and a more critical engagement with its own limitations. With revisions, it has strong potential to contribute significantly to literature and policy on child nutrition in Sub-Saharan Africa.
