# Supplementary material for: Unveiling the full picture of malnutrition in Sub-Saharan Africa: The extended composite index of anthropometric failure among children under-5 in the SDG era
Source: PLoS One. 2025 Oct 3;20(10):e0333785. doi: 10.1371/journal.pone.0333785 (PMC12494244; doi:10.1371/journal.pone.0333785)
Supplement: Attachment — Submitted filename: Reviewer comment_Plos One.docx [file pone.0333785.s004.docx]

| Title | The title is **too long** and contains **two colons,** that affects readability |
| --- | --- |
|  | The term “Extended” is somewhat ambiguous—it’s unclear whether it refers to an enhancement of the Composite Index of Anthropometric Failure (CIAF) itself or to a broader scope of analysis.  If it denotes a novel or modified version of the index, this should be explicitly stated, such as by using phrasing like “a novel extension of the CIAF. |
| Abstract | *Terminology and consistency: there is inconsistency in how some terms are presented—e.g., “children unde-5 years old” (typo) and variations in phrasing (e.g., “under-five,” “under 5,” “under-5”) should be standardized.  *(Result, line 46) Language and Grammar: revise run-on sentences, awkward phrasing such as “child, maternal and household and community level characteristics”), which need editing for clarity and fluency.  *Redundancy and Repetition**:** There is **repetition** of associated factors in both the Results and Conclusions sections (e.g., child sex, birth interval, maternal education, etc.), which could be streamlined, it dilute the impact of the key findings.  *Statistical Reporting: mentioning specific adjusted odds ratios (for key predictors) would further strengthen the statistical insight. |
| Introduction | Line 70-73: how ECIAF improves upon previous indices beyond just integration (e.g., is it validated? more predictive?). |
|  | Line 88-94Please add real-world example of a child being both stunted and wasted to add clarity and relevance. |
|  | Link to global goals: Lines 85–87: the sentence “The ECIAF aligns with these global objectives…” could benefit from stronger evidence or citation supporting its alignment or usage in global or national planning.  **Line 95**: “Grounded in a comprehensive review…” is a **strong but unsubstantiated claim.** It would be helpful to briefly indicate how ECIAF was developed or cite the source. |
|  | Policy & practical relevance: Lines 107–112: briefly reference where ECIAF has been applied in decision-making or implementation. |
|  | Lines 113–119: Phrases like “several advantages” a bit generic it should be specified. Examples of use by NGOs or in surveys would reinforce the argument.  The advantages are reiterated in multiple places (e.g., lines 72–73, 80–81, 107–109, 120–123), and could be more concisely synthesized. |
|  | Revise **vague phrasing (**e.g., “providing a nuanced understanding,” line 72; “true extent of nutritional deficiencies,” line 93). These could be replaced with more precise language or examples. |
|  | **Lines 120–127**: it risks **overstating** its impact without acknowledging **potential limitations,** such as data requirements, complexity in computation, or need for validation. |
| Methods | Line 131: the phrase "in the sustainable development era" is vague. Clarify **what year range** this refers to (e.g., "DHS surveys conducted between 2015 and 2022"). |
|  | line 134: Sierra Leon should be corrected to Sierra Leone. |
|  | Line 132-135: Consider moving the list of countries to a supplementary table or referencing it in a table/appendix to improve readability and flow. |
|  | Sampling methodology: Line 138-141: explanation is **overly generic**. Since this is a secondary data analysis, clarify **whether sample weights** were applied in the pooled analysis and how **country-level differences** (e.g., survey year, population size) were addressed. |
|  | Data used (line 142-143): more detail is needed on **inclusion/exclusion criteria** (e.g., were only children with complete anthropometric data included? Were outliers or biologically implausible values excluded?). |
|  | Line 143: Make the total sample size reported accounts for **valid and complete cases** only**.** |
|  | **Lines 144–146:** in addition to the link to the DHS methodology good to **cite one or two standard DHS methodology references** (e.g., Rutstein and Rojas, DHS Comparative Reports). |
|  | **Lines 149–157 and 186–199**: ECIAF is described in multiple places, with some **redundancy**. These parts should be **merged and streamlined**. |
|  | **Line 150**: Typo—“W/H>22 Z-score” should likely be “W/H > +2 Z-score” (or BMI/A > +2 SD). |
|  | **Line 152**: The sentence "subtracting children without anthropometric failure...from the sum..." is confusing. Mathematically, this explanation doesn’t make sense. What is meant is likely that **ECIAF = 1 if a child falls into any failure group (B to H), otherwise 0.** |
|  | Check font type consistency: line 161 to 168 |
|  | * General: Add the survey years for each country or the range of years to understand temporal comparability.  *Consider including how missing data were handled and whether any adjustments were made to account for survey design effects (e.g., use of Stata svy commands or multilevel modeling).  *Mention data use permission, to demonstrate compliance with data governance standards. |
|  | **Lines 190–198**: Several category labels are inconsistently capitalized (e.g., underweight and wasting vs. wasting and underweight), and the **sequence of labels (F, Y, G, H)** is not logically arranged. Also, “F: stunting only” is described differently in the narrative than in the table, **ensure consistency**. |
|  | **Lines 200–202**: The table is useful, but formatting needs improvement for clarity (e.g., align headers, avoid text breaking mid-sentence). Add a **proper caption** and clarify all acronyms (e.g., DE likely means SD). |
|  | ECIAF calculation line (**line 200**) is imprecise. It should be:  ECIAF = % of children falling into any of the categories B through H. |
|  | **Terminology**: Avoid inconsistent phrasing like “community women education” (line 167); revise to “community-level women’s education.” |
|  | **Lines 160–172**: Some variables (e.g., media exposure, wealth index) are repeated or categorized inconsistently across lines. |
|  | **Lines 174–177**: Appears to be a continuation of individual-level variables but is disconnected from prior sentences**,** integrate **and structure this list more clearly.** |
|  | **Line 203–209**: Good to define Africa regions, but this information might be better placed in the Methods or Supplementary Information, not under “Measurement variables.” |
|  | **Lines 210–212** (Community poverty) and 213–215 (Community literacy): the method of aggregating and dichotomizing the variables (e.g., "median split") may oversimplify continuous variation and should be justified in analysis or discussion. |
|  | **Line 217**: "Cleaned, appended, recoded" is vague. Specify **what variables were recoded, how missing data were handled, and criteria for data cleaning or exclusion.** |
|  | **Line 219**: The sentence is poorly structured—"before the analysis the by using" needs correction. Suggested rewrite:  "Data were weighted prior to analysis using sampling weights, primary sampling units, and stratification variables to ensure national representativeness." |
|  | **Lines 232–234** repeat what was already stated earlier (lines 219–220). This redundancy should be eliminated or merged into one cohesive paragraph. |
|  | **Line 233**: Make clear **how weighting was implemented** in the multilevel regression (e.g., using svy commands, or incorporated into mixed models). |
|  | Lines 221–224: Clarify the levels of the model (e.g., children nested within clusters or countries?).  *****Specify whether random intercept or random slope models were used. |
|  | **Line 224**: “Bivariable” should be corrected to **"bivariate”** |
|  | **Lines 225–227**: The variable selection process (p < 0.2, literature, and clinical expertise) is reasonable, but **"clinical expertise"** is ambiguous in secondary data analysis. Clarify whether domain experts were consulted or if prior empirical findings guided this. |
|  | Lines 228–243: Some definitions are copied twice (see lines 228–230 and 236–243), which is redundant.  *****Formula for ICC (line 240) is correct but should be introduced with clarity.  *Define PCV (Proportional Change in Variance)—it's mentioned in line 229 but never explained. |
|  | **Line 239**: "WRA" (women of reproductive age) seems out of context here. This study focuses on children under five, so this may be an **editing error.** |
|  | Lines 244–249: Clarify whether random effects were included in all models, and how the final model controlled for country- or cluster-level heterogeneity.  *The use of log-likelihood ratio and deviance is appropriate. You may consider also reporting AIC/BIC for model comparison. |
|  | **Line 253**: “clustrs” is a typo; should be “clusters |
|  | Lines 255–260: You may **explicitly mention** that permission to use DHS data was obtained via registration on [DHS program website](https://dhsprogram.com). |
| Result | Lines 262–270: Study population characteristics: Line 268: Change "Northern African countries" to "Northern African country" to match singular form (only Mauritania). |
|  | Line 273: “After recoded of the child age majorities” is grammatically awkward. Consider: “After recoding child age, the majority (57.09%) were 24–59 months…” |
|  | Lines 271–278: Basic descriptive statistics: Inconsistent use of past and present tense throughout. Use **past tense** consistently for reporting survey results.  *Consider specifying “weighted percentage” for clarity when presenting weighted data. |
|  | Lines 279–278: Table 2: Line 274: “nearly three-fourth (64.09%)” → should be “nearly two-thirds |
|  | Line 275: Typo: “64.09% of were in children…” → remove “of” or rewrite for clarity. |
|  | Lines 281–288: Prevalence of ECIAF by characteristics: Consider using Pearson’s Chi-square p-values in Table 3 to denote statistically significant differences in ECIAF prevalence by characteristics.  * Use consistent formatting for n (%) across all entries. |
|  | Lines 290–300: Pooled prevalence: Line 293: “Burundi 59%… and Gabon 20%…” → Reword to: “Prevalence ranged from 59% in Burundi (95% CI: 58–60%) to 20% in Gabon (95% CI: 19–21%).”  * “Figure 1” should be referenced properly with an in-text caption and axis explanation. |
|  | Lines 301–319: Subgroup analysis by region: Line 310: “Sothen Africa” should be corrected to “Southern Africa”.  * Sentence structures in Lines 304–314 are repetitive and sometimes unclear. Suggested revision: Present findings in bullet points or tabular format to avoid long sentences.  * Line 309: “lowest both countries…” is grammatically incorrect. Consider: “The lowest prevalence was observed in Ghana and Gambia (both 24%).” |
|  | Lines 320–368: Fixed effect analysis:  *Use consistent terminology: “higher odds of ECIAF” or “increased risk” do not alternate between both in the same paragraph.  *Clarify the reference categories in-text where not obvious (e.g., for household size, parity, education).  * Line 342: “has comorbidity” → revise to “had comorbidity”.  *Line 348: Minor grammatical issue: “has no antenatal care visit” → revise to “did not have ANC visits”.  *Line 357: “3–5 under-5 children” is confusing—does it mean 3 to 5 under-5s or 3 to 5 children total? Clarify. |
|  | **Lines 369–372: Table 4**: Table 4 needs to be formatted carefully and consistently across models. Some AORs have inconsistent spacing or punctuation.  * Table title (Line 370) should specify that results are from a multilevel logistic regression. |
|  | Lines 374–389: Random effect analysis and model fit statistics: Line 381: Typo in parenthesis – “i.e one from a cluster…” → “i.e., one from a cluster…”  Line 385: “63.58% of the variability in the ECIAF…” → “of the variability in ECIAF” (drop “the”). |
|  | Lines 390–392: Table 5: Table 5 title says “in Ethiopia” (Line 392), but this is a multi-country study. Correct to “in Sub-Saharan Africa”. |
| Discussion | Lines 395–400: Introduction of findings: Line 400: Awkward phrasing: “To compare the finding…there was no similar research…” → Recommend: “There is limited prior research using ECIAF in the same context; however, our findings align with CIAF-based studies.” |
|  | Lines 401–405: Comparison with other studies: But, this prevalence aligns…” → Remove “But,” for better flow. |
|  | Line 404: Avoid starting a sentence with “While” when not showing contrast. Suggest: “A study in Bangladesh reported a CIAF prevalence of 48.3%...” |
|  | Lines 406–415: Regional disparities: Line 411: “ranging from 44% in Angola to 38% in South Africa” – the wording is fine, but the contrast is weak. Consider: *“...with Angola (44%) reporting the highest, and South Africa (38%) the lowest within the region.”*  * Line 415: “Male children were more affected…” → Good point, but link to mechanism or literature earlier. |
|  | Lines 416–424: Multilevel findings overview: Line 424: End punctuation is a misplaced superscript “33.” Should either be removed or properly cited. |
|  | Lines 425–437: Detailed associations: **Split this into subheadings or separate paragraphs** (e.g., child-level, maternal, household, community factors).  * Line 426: “child birth average size, small size…” → redundant. Suggest condensing.  * Line 430: “and negatively were institutional delivery…” → revise: “...and were negatively associated with ECIAF: institutional delivery, ANC visits, and grand multiparity.”  * Avoid repeating “positively associated with the risk…” in every clause. Group ideas logically. |
|  | Lines 438–452: Interpretations: *Line 446: “short birth intervals (<24 months) also had greater risk” → “also carried greater risk”  *Line 448–450: “Perceived birth size…” and “corroborating evidence…” – Good use of literature. |
|  | Lines 453–460: Maternal characteristics: Line 459: Consider clarifying how “grand multiparity” being protective differs from what’s found in other literature. |
|  | Lines 461–467: Household characteristics: Line 466: “Conversely, larger family sizes…” – Provide potential mechanism briefly.  * Consider distinguishing between family size and number of under-5s for clarity. |
|  | Lines 468–477: Community-level and regional factors: Line 475: “Southern, Western, Eastern, and Central Africa…” → Consider reordering by prevalence or alphabetically. |
|  | Lines 478–483: Implications: Slight repetition with applications section. Consider integrating or reducing overlap. |
|  | Lines 484–496: Applications: Line 486: Start with stronger topic sentence: “The findings have several practical implications for child health policy and programming.”  * Line 494: “wealth redistribution strategies” – might be too politically charged for some journals; consider: “economic empowerment initiatives”. |
|  | Lines 497–503: Health system: Avoid overly long sentences (e.g., Line 499–500).  *Consider mentioning health workforce training or integration of nutrition into routine services. |
|  | Lines 504–509: Community-based programs: Could benefit from examples or references from successful case studies. |
|  | Lines 510–516: Research: Line 513: “Longitudinal studies…” – clarify why they are particularly important (e.g., to establish causality). |
|  | Line 517: Wrap-up Sentence: Incomplete sentence due to cutoff. Consider completing as: “...and guide future programming to reduce child malnutrition in Sub-Saharan Africa.” |
