# Supplementary material for: Unveiling the full picture of malnutrition in Sub-Saharan Africa: The extended composite index of anthropometric failure among children under-5 in the SDG era
Source: PLoS One. 2025 Oct 3;20(10):e0333785. doi: 10.1371/journal.pone.0333785 (PMC12494244; doi:10.1371/journal.pone.0333785)
Supplement: Attachment — Submitted filename: Response to reviewers.docx [file pone.0333785.s005.docx]

**Date: July 31, 2025**

**Rebuttal letter**

**Response to the journal requirements questions**

**Question #1.**

Please ensure that your manuscript meets PLOS ONE's style requirements, including those for file naming. The PLOS ONE style templates can be found at

<https://journals.plos.org/plosone/s/file?id=wjVg/PLOSOne_formatting_sample_main_body.pdf> and

[https://journals.plos.org/plosone/s/file?id=ba62/PLOSOne_formatting_sample_title_authors_affiliations.pdf](https://track.editorialmanager.com/CL0/https:%2F%2Fjournals.plos.org%2Fplosone%2Fs%2Ffile%3Fid=ba62%2FPLOSOne_formatting_sample_title_authors_affiliations.pdf/1/010f0198587f8856-80b2524a-6435-4e50-b441-139156c8fbb0-000000/Aj1jMo3AkhIdDxdghCCdnXY6ZBcEK6dQDwvMpjG0E6s=222)

**Response:** Thank you for this remark. We formatted the manuscript using the PLoS ONE format guidelines. The whole content of the manuscript, including the abstract, introduction, methods, results, discussion and references are formatted using the guidelines (**please see the revised version for each section**).

**Question #2.**

We note that your Data Availability Statement is currently as follows: All relevant data are within the manuscript and in Supporting Information files.

Please confirm at this time whether or not your submission contains all raw data required to replicate the results of your study. Authors must share the “minimal data set” for their submission. PLOS defines the minimal data set to consist of the data required to replicate all study findings reported in the article, as well as related metadata and methods (<https://journals.plos.org/plosone/s/data-availability#loc-minimal-data-set-definition>).

For example, authors should submit the following data:

- The values behind the means, standard deviations and other measures reported;

- The values used to build graphs;

- The points extracted from images for analysis.

Authors do not need to submit their entire data set if only a portion of the data was used in the reported study.

If your submission does not contain these data, please either upload them as Supporting Information files or deposit them to a stable, public repository and provide us with the relevant URLs, DOIs, or accession numbers. For a list of recommended repositories, please see <https://journals.plos.org/plosone/s/recommended-repositories>.

If there are ethical or legal restrictions on sharing a de-identified data set, please explain them in detail (e.g., data contain potentially sensitive information, data are owned by a third-party organization, etc.) and who has imposed them (e.g., an ethics committee). Please also provide contact information for a data access committee, ethics committee, or other institutional body to which data requests may be sent. If data are owned by a third party, please indicate how others may request data access.

**Response:** All relevant data necessary to replicate the study’s findings are available within the manuscript and its Supporting Information files. The dataset used in this analysis is derived from the Demographic and Health Surveys (DHS) Program, which is a publicly accessible, third-party source. Due to data ownership and usage restrictions, we are not permitted to share the raw DHS dataset directly. However, the minimal data set underlying the results of this study—including the variables used, coding details, and values behind reported means, percentages, and regression analyses—are provided in the Supporting Information files.

Researchers can obtain the original DHS data by registering for free and submitting a request through the DHS Program website: https://dhsprogram.com/data/. Access is granted to qualified researchers upon approval by the DHS Program. Data requests may be directed to:
**The DHS Program**

ICF, 530 Gaither Road, Suite 500, Rockville, MD 20850, USA

Email: **dhsprogram@icf.com**

**Question #3.**

Please ensure that you refer to Figure 1 and 2 in your text as, if accepted, production will need this reference to link the reader to the figure.

**Response:** We have ensured the figures are appropriately cited.

**Question #4.**

If the reviewer comments include a recommendation to cite specific previously published works, please review and evaluate these publications to determine whether they are relevant and should be cited. There is no requirement to cite these works unless the editor has indicated otherwise.

**Response:** We have reviewed all recommendations of the editor and feedbacks of all reviewers, assessed their relevance to our study, and took actions which could strengthen the manuscript.

**Line-by-line response to reviewers**

**Reviewer #1**

**Comment #1.**

**Title of Manuscript**: *Unveiling the full picture of malnutrition: Extended composite index of anthropometric failure among children under-5 in Sub-Saharan Africa: In the SDG era*

This manuscript presents a comprehensive analysis of child malnutrition in Sub-Saharan Africa, focusing on the Extended Composite Index of Anthropometric Failure (ECIAF). The study explores the prevalence and regional disparities of ECIAF among children under five years old, offering insights into the multifaceted nature of malnutrition. By examining key determinants at individual, maternal, household, and community levels, the authors provide a nuanced understanding of the factors contributing to childhood malnutrition in the context of the Sustainable Development Goals (SDGs). This research is timely and relevant, addressing a pressing public health issue and proposing actionable policy recommendations. However, I have some comments to improve the manuscript as follows:

**Introduction Section:**

- **The introduction clearly contextualizes the significance of the study and the relevance of ECIAF in addressing child malnutrition. However, there is room for improvement in the transition between traditional indices and ECIAF. Adding a linking sentence between these two concepts would enhance the overall flow.**

**Response**: Thank you very much for your kind feedbacks. We updated the manuscript. **Please see the revised manuscript.**

- The explanation of why traditional indices fall short in capturing the multifaceted nature of malnutrition could be clarified further.

**Response**: Traditional indices such as weight-for-age (underweight), height-for-age (stunting), and weight-for-height (wasting) have long been used to assess child malnutrition. However, these indicators measure separate dimensions of malnutrition in isolation and do not account for their possible overlap within the same child. For instance, a child may be both stunted and underweight, or simultaneously wasted and stunted — scenarios that traditional measures cannot adequately reflect. This fragmented approach may underestimate the true burden of malnutrition and limit the effectiveness of targeted interventions.

- **Line 69-71**: Clarify why traditional indices fall short in capturing the full scope of malnutrition. Consider revising: "Traditional indices such as weight-for-age, height-for-age, and weight-for-height have been instrumental in monitoring child nutrition, but they often fail to capture the multifaceted nature of malnutrition."

**Response**: “Traditional indices such as weight-for-age (underweight), height-for-age (stunting), and weight-for-height (wasting) have been valuable in assessing specific types of malnutrition. However, they evaluate these conditions separately, failing to account for the simultaneous occurrence of multiple forms of anthropometric failure in the same child. As a result, these indices underestimate the overall burden of malnutrition and do not fully reflect its complexity.”

- **Line 74-77**: Add references to support claims about long-term health outcomes related to malnutrition. Also, refine the sentence for clarity: "Malnutrition in children under five includes stunting (low height-for-age), wasting (low weight-for-height), and underweight (low weight-for-age), all of which indicate immediate nutritional deficiencies."

**Response**: The Manuscript is updated. **Please see the revised manuscript.**

- **Line 101-106**: Simplify the phrasing: "absence of a standardized composite measure" rather than "lack of a standardized composite measure."

**Response**: The Manuscript is revised. **Please see the revised manuscript.**

- **Line 82**: Strengthen the paragraph discussing the SDGs by linking ECIAF’s relevance directly to these goals.

**Response**: The Manuscript is revised. **Please see the revised manuscript.**

- **General suggestion**: Strengthen the introduction by explicitly stating how ECIAF can contribute to achieving the SDGs and can serve as a transformative tool in addressing malnutrition in Sub-Saharan Africa.

**Response**: We have updated the Manuscript. **Please see the updated manuscript.**

**Comment #2.**

**Methods Section:**

- The description of the two-stage stratified cluster sampling is well-done but could benefit from additional details, particularly on how strata were defined (e.g., by region, urban/rural). This would improve the understanding of the sample's representativeness.

**Response**: The manuscript is updated. **Please see the updated manuscript.**

- **Sampling Procedure**: Add more detail on how strata were defined to clarify how different regions or population groups were represented.

**Response**: The manuscript is updated. **Please see the updated manuscript.**

- **Data Collection Period**: Include the specific period during the SDG era when the data was collected. This is important to provide context for the results.

**Response**: The manuscript is updated. **Please see the updated manuscript.**

**Statistical Methods**:

- A brief explanation of why multilevel binary logistic regression was chosen would help clarify its relevance.

**Response**: Multilevel binary logistic regression was employed to account for the hierarchical structure of the DHS data, where individual children (level 1) are nested within clusters or communities (level 2), such as Enumeration Areas (EAs). This modeling approach was chosen because it adjusts for intra-cluster correlation and allows for the simultaneous estimation of both individual-level and community-level determinants of extended composite index of anthropometric failure (ECIAF). Without accounting for this clustering, standard errors could be underestimated, leading to inflated Type I error rates. Multilevel modeling thus provides more accurate estimates and enhances the validity of inferences drawn from the data.

- Consider including **sensitivity analysis** or robustness checks to strengthen the validity of the results.

**Response**: To assess the robustness of the findings, sensitivity analyses were conducted by (1) restricting the sample to countries with DHS data collected within the most recent five years (e.g., 2017–2022), and (2) excluding countries with extreme ECIAF prevalence (e.g., Burundi and Gabon). The multilevel binary logistic regression was re-estimated under these alternative specifications to evaluate whether key associations with ECIAF remained consistent.

**General suggestions**:

- **ECIAF Definition**: Clarify the categories used in the ECIAF calculation (Groups A-H). Providing a clearer and more structured presentation will improve readability.

**Response**: The manuscript is updated. **Please have a look at on it again.**

- **Statistical Model Details**: Add a brief explanation of the statistical model fitting procedure (e.g., likelihood ratio tests, AIC) to improve transparency.

**Response**: The manuscript is updated. **Please have a look at on it again.** But writing every think in the manuscript makes bulky.

**Comment #3.**

**Results Section:**

- The results section does a good job presenting the ECIAF prevalence. However, phrasing can be improved for clarity. For instance, instead of “the prevalence of ECIAF was 36% (95% CI: 33%, 40%)”, it could be reworded for better readability.
- **Phrasing**: "The overall pooled prevalence of ECIAF in children under five in Sub-Saharan Africa was 36%, with a 95% confidence interval ranging from 33% to 40%."

**Response**: The Manuscript is updated. Please see it the final version.

- **Subgroup Analyses**: The descriptions of subgroup prevalence rates (e.g., for East Africa, West Africa) could be better organized. For example, when presenting the regional differences in prevalence, include clearer transitions and structure.

**Response**: The Manuscript is updated. Please see it the final version.

- **Odds Ratios**: Provide a brief interpretation of odds ratios (AORs) for non-statistical readers. Explain their practical implications for public health.

**Response:** We have tried to correct the comments.

**Comment #4.**

**Discussion Section:**

- Some sentences are complex and could benefit from simplification. For example, the list of factors associated with ECIAF in children under 5 years could be broken down into clearer subpoints.
- **Sentence Structure**: Break down complex sentences like "The risk of having ECIAF was positively associated with child characteristics..." into more digestible parts.
- **Policy Implications**: The discussion could more strongly emphasize the **public health implications** of these findings. Specifically, consider elaborating on how the results can inform policy changes related to maternal education, child health, and nutrition.

**Response:** The manuscript is updated. **Please see the revised version of the manuscript.**

**Comment #5.**

**Implications, Applications, Limitations, and Conclusions:**

**Implications**:

- The implications are well-articulated but can be made more concise to improve readability. For example, consider simplifying the phrasing: "Strengthening maternal health services, promoting family planning, improving socioeconomic conditions, and enhancing access to health information through media" could be shortened to "Strengthening maternal health services, improving family planning, and promoting socio-economic development."

**Response:** The manuscript is updated. **Please see the revised version of the manuscript.**

**Applications**:

- While the applications are comprehensive, the section would benefit from linking the findings more explicitly to specific interventions. Consider adding a sentence that connects the study’s results to practical applications.
  - Suggested revision: "The findings highlight the need for targeted interventions, such as improving maternal education and increasing access to healthcare, to address ECIAF."

**Response:** The manuscript is updated. **Please see the revised version of the manuscript.**

**Limitations**:

- The limitations section appropriately acknowledges the cross-sectional nature of the study. However, it could be strengthened by discussing potential measurement biases from secondary data sources and missing variables like media exposure and distance to healthcare facilities.
- **Future Research**: Suggest that future research could overcome these limitations by using longitudinal designs and exploring additional factors like healthcare access and social determinants of health.

**Response:** The manuscript is updated. **Please see the revised version of the manuscript.**

**Conclusions**:

- The conclusions effectively summarize the findings, but could benefit from a stronger emphasis on **policy implications**. For example, suggest specific actions such as improving maternal education and providing targeted nutrition programs for vulnerable populations.

**Response:** The manuscript is updated. **Please see the revised version of the manuscript.**

**Reviewer #2**

**Comment #1.**

**Title and Abstract**

**Strengths:**

The title is descriptive and informative, clearly reflecting the study’s aim and population of interest.

The abstract concisely presents the background, methods, results, and conclusions.

**Areas for improvement:**

- The abstract could better reflect the novel aspects of the Extended Composite Index of Anthropometric Failure (ECIAF), possibly with a brief explanation of how it surpasses traditional measures.

**Response:** Thank you for the positive assessment of our work and your well-articulated feedback is highly appreciated. We updated the manuscript.

- The use of acronyms like ECIAF could be confusing to non-technical readers in the abstract; consider spelling it out first.

**Response:** We updated the manuscript. **Please see the revised version of the manuscript.**

**Comment #2.**

**Introduction**

**Strengths:**

The introduction is well-grounded in global health goals (SDGs) and articulates the importance of accurate malnutrition assessment.

It outlines the rationale for using ECIAF and connects it to policy relevance.

**Areas for improvement:**

- The literature review could be more current, with clearer referencing to similar studies post-2020.

**Response:** As to our search, we couldn’t find similar studies done post-2020. We have conducted a comprehensive search using different databases and search engines. But we tried to include recent studies.

- The justification for choosing ECIAF over other composite measures could be better articulated with empirical comparisons.

**Response:** The manuscript is updated. Please see the revised version of the manuscript.

**Comment #3.**

**Methods**

**Strengths:**

The use of a large, representative dataset across 26 Sub-Saharan African countries is commendable.

The stratification into individual- and community-level factors and use of multilevel modeling enhance robustness.

**Areas for improvement:**

- The operational definition of ECIAF, while technically accurate, is overly dense. A simplified diagram or table might help.

**Response:** We revised the manuscript by reorganizing the operational definition for ECIAF. **Please see the revised manuscript.**

- More detail is needed on ethical considerations, especially since secondary data use still carries responsibility for safeguarding privacy.

**Response:** We have updated the ethical consideration section. **Please see the revised manuscript.**

- The variable selection process (p-value < 0.2 + literature + expert judgment) is common but should be justified more explicitly for transparency.

**Response:** Yes, the variable selection is based on the statistical, clinical and literature review. If we write every step of the procedure, the manuscript becomes bulk. To avoid unnecessary words, we have written the very important issues.

**Comment #4.**

**Results**

**Strengths:**

Clear presentation of descriptive data, subgroup analysis, and multivariate findings.

Use of figures and tables helps to break down complex results.

**Areas for improvement:**

The writing could be more fluid; it reads as a data dump in parts. Focus on highlighting trends, not just numbers.

Regional variations are well-presented, but the manuscript does not delve deeply into why these differences exist—more interpretive insight is needed.

**Response:** We re-write the result section as your suggestion provided to us. **Please check the revised manuscript.**

**Comment #5.**

**Discussion**

**Strengths:**

The discussion covers multiple determinants of ECIAF and links findings to prior research.

There’s a commendable effort to draw policy implications from the data.

**Areas for improvement:**

- There’s repetition from the results section; consider condensing and focusing on interpretation.

**Response:** We have revised the manuscript.

- The discussion lacks a critical reflection on the study's limitations. For example, potential bias from self-reported birth sizes or unmeasured confounders should be acknowledged.

**Response:** As per the feedbacks from the reviewer, we have revised the manuscript.

- The regional comparison could benefit from linking findings to historical, cultural, or policy-based explanations.

**Response:** The manuscript is updated. **Please see the final version of the manuscript.**

**Comment #6.**

**Conclusion**

**Strengths:**

It concisely summarizes key findings and policy relevance.

Emphasizes practical interventions such as improving maternal education and health access.

**Areas for improvement:**

- A clearer statement on the novelty of ECIAF and its potential for replication across other regions would strengthen the final impact.

**Response:**

- A recommendation for future research directions (e.g., longitudinal studies or validation of ECIAF) would add value.

**Response:** We have corrected right now. **Please check the revised version of the manuscript.**

**Comment #7.**

**Language and Structure**

**Strengths:**

Generally readable and logically structured.

Tables and figures are appropriate and informative.

**Areas for improvement:**

- Grammatical inconsistencies and typos (e.g., “unde-5” instead of “under-5,” “Sothen Africa”) should be corrected for professionalism.

**Response:** We have addressed the issue to our best.

- Some paragraphs are too long or overly technical; consider simplifying for broader audiences.

**Response:** We tried to revise the whole parts of the manuscript.

**Comment #8.**

**Overall Assessment**

This manuscript tackles an important public health issue with methodological rigor and a large dataset. The ECIAF provides an innovative approach to understanding malnutrition beyond standard indices. However, the manuscript would benefit from clearer articulation of novelty, improved interpretation of regional differences, and a more critical engagement with its own limitations. With revisions, it has strong potential to contribute significantly to literature and policy on child nutrition in Sub-Saharan Africa.

**Response:** Dear reviewer, we highly appreciate your investing your time and feedbacks for the improvement of the manuscript. We have tried to improve the whole parts of the manuscript to contribute novel idea and rigour methodology to the scientific community and future research. **Please check the revised manuscript.**

**Reviewer #3**

**Comment #1.**

Title

- The title is **too long** and contains **two colons,** that affects readability

**Response:** Dear reviewer, we thank you very much for your detailed review, genuine feedbacks and time you invest for the improvement of our manuscript. We modify the title somewhat a little. **Please see the updated version of the manuscript.**

- The term “Extended” is somewhat ambiguous—it’s unclear whether it refers to an enhancement of the Composite Index of Anthropometric Failure (CIAF) itself or to a broader scope of analysis. If it denotes a novel or modified version of the index, this should be explicitly stated, such as by using phrasing like “a novel extension of the CIAF.

**Response:** We appreciate your concerns on the title. The title is a novel extension of the CIAF. But, is better to be described by the term “extended” i.e why the ECIAF incorporates “weight excess (being overweight or obese)”.

**Comment #2.**

Abstract

*Terminology and consistency: there is inconsistency in how some terms are presented—e.g., “children unde-5 years old” (typo) and variations in phrasing (e.g., “under-five,” “under 5,” “under-5”) should be standardized.

**Response:** It has been corrected throughout the manuscript right now.

*(Result, line 46) Language and Grammar: revise run-on sentences, awkward phrasing such as “child, maternal and household and community level characteristics”), which need editing for clarity and fluency.

**Response:** We have corrected it.

*Redundancy and Repetition**:** There is **repetition** of associated factors in both the Results and Conclusions sections (e.g., child sex, birth interval, maternal education, etc.), which could be streamlined, it dilute the impact of the key findings.

**Response:** We have revised it.

*Statistical Reporting: mentioning specific adjusted odds ratios (for key predictors) would further strengthen the statistical insight.

**Response:** We have updated. Please check the updated submission.

**Comment #3.**

Introduction

Line 70-73: how ECIAF improves upon previous indices beyond just integration (e.g., is it validated? more predictive?).

**Response:** The ECIAF offers a more comprehensive and validated measure by integrating multiple forms of anthropometric failure and capturing their overlap. Beyond simple aggregation, it reveals the full extent of malnutrition burden, reduces underestimation, and provides a more sensitive tool for identifying at-risk children which enhances its utility for public health planning, monitoring, and policy-making.

Line 88-94: Please add real-world example of a child being both stunted and wasted to add clarity and relevance.

**Response:** Previous research has extensively documented the prevalence and determinants of malnutrition using traditional indices. However, these studies often overlook the simultaneous occurrence of multiple forms of malnutrition in the same child. For instance, a child may be both stunted: indicating chronic undernutrition: and wasted: reflecting acute weight loss. In one real-world case from rural Ethiopia, a 24-month-old girl presented with a height-for-age z-score of -2.8 and a weight-for-height z-score of -2.6. According to traditional indices, she would be counted either as stunted or wasted depending on the reporting framework: but not both. ECIAF captures such overlapping conditions, thereby revealing the true extent of nutritional deficiency and ensuring no child is overlooked in policy and programming (7–9).

Link to global goals: Lines 85–87: the sentence “The ECIAF aligns with these global objectives…” could benefit from stronger evidence or citation supporting its alignment or usage in global or national planning.

**Response:** The ECIAF aligns with global objectives such as SDG 2 (Zero Hunger) and SDG 3 (Good Health and Well-being) by offering a comprehensive measure of child malnutrition that captures its multidimensional nature. While ECIAF is not yet part of official global monitoring frameworks, studies have recommended its adoption in national health assessments due to its ability to uncover a higher burden of malnutrition than traditional indicators (Bejarano et al., 2019; Ayres et al., 2024). Its growing application in countries like Ethiopia and Bangladesh demonstrates its potential to support more targeted planning, resource allocation, and intervention design in line with global nutrition goals.

**Line 95**: “Grounded in a comprehensive review…” is a **strong but unsubstantiated claim.** It would be helpful to briefly indicate how ECIAF was developed or cite the source.

**Response:** The development of the ECIAF was proposed by Bejarano et al. (2019) through a critical evaluation of traditional anthropometric indicators and their limitations in capturing overlapping malnutrition conditions. Drawing on anthropometric data from multiple national health surveys and previous composite index models such as the CIAF, the ECIAF was designed to reflect the full spectrum of undernutrition and overweight conditions in a single, more inclusive measure (9).

Policy & practical relevance: Lines 107–112: briefly reference where ECIAF has been applied in decision-making or implementation.

**Response:** The health policy relevance of the ECIAF cannot be overstated. By providing a more accurate and comprehensive measure of child malnutrition, this index can inform the design and implementation of targeted nutrition programs. For example, ECIAF has been used in national analyses in Ethiopia and Bangladesh to identify regional disparities and prioritize areas for intervention (Islam & Biswas, 2020; Ayres et al., 2024). Although it has not yet been formally adopted into DHS or WHO reporting standards, its growing application in research and program evaluations highlights its value for policymakers seeking to allocate resources more effectively and monitor complex malnutrition patterns.

Lines 113–119: Phrases like “several advantages” a bit generic it should be specified. Examples of use by NGOs or in surveys would reinforce the argument.

**Response:** From a practical standpoint, the ECIAF offers **distinct advantages** over traditional indices by capturing the full spectrum of anthropometric failures — including children with multiple overlapping conditions who might otherwise be missed. For instance, it enables **more precise targeting** of nutrition interventions by identifying the most vulnerable subpopulations. In Ethiopia, researchers have used ECIAF in DHS-based analyses to inform regional prioritization of child nutrition programs (Ayres et al., 2024). Similarly, in Bangladesh, ECIAF has guided local NGOs such as BRAC in identifying dual burdens of undernutrition for programmatic planning (Islam & Biswas, 2020). Community health workers and monitoring agencies benefit from ECIAF’s ability to consolidate complex nutritional data into a **single, actionable metric**, making it easier to track intervention impact across diverse settings.

The advantages are reiterated in multiple places (e.g., lines 72–73, 80–81, 107–109, 120–123), and could be more concisely synthesized.

**Response:** It is revised now.

Revise **vague phrasing (**e.g., “providing a nuanced understanding,” line 72; “true extent of nutritional deficiencies,” line 93). These could be replaced with more precise language or examples.

**Response:** Replaced with a more precise language.

**Lines 120–127**: it risks **overstating** its impact without acknowledging **potential limitations,** such as data requirements, complexity in computation, or need for validation.

**Response:** We have revised based on the feedback.

**Comment #4.**

Methods

Line 131: the phrase "in the sustainable development era" is vague. Clarify **what year range** this refers to (e.g., "DHS surveys conducted between 2015 and 2022").

**Response:** Dear reviewer, we have made correction on your concern.

line 134: Sierra Leon should be corrected to Sierra Leone.

**Response:** We have edited the manuscript. **Please look at on the revised version of the main manuscript.**

Line 132-135: Consider moving the list of countries to a supplementary table or referencing it in a table/appendix to improve readability and flow.

**Response:** We have taken it to the supplementary file in table format.

Sampling methodology: Line 138-141: explanation is **overly generic**. Since this is a secondary data analysis, clarify **whether sample weights** were applied in the pooled analysis and how **country-level differences** (e.g., survey year, population size) were addressed.

**Response:** Yes, it is a secondary data analysis. We considered a sample weights for each country. It is described elsewhere.

Data used (line 142-143): more detail is needed on **inclusion/exclusion criteria** (e.g., were only children with complete anthropometric data included? Were outliers or biologically implausible values excluded?).

**Response:** Children aged 0–59 months were included in the analysis if they had complete and valid anthropometric measurements for height, weight, and age, enabling the computation of weight-for-age (WAZ), height-for-age (HAZ), and weight-for-height (WHZ) Z-scores. Following WHO recommendations, biologically implausible values (BIVs) for Z-scores (e.g., HAZ < -6 or > +6, WHZ < -5 or > +5, WAZ < -6 or > +5) were excluded from the analysis.

Line 143: Make the total sample size reported accounts for **valid and complete cases** only**.**

**Response:** Yes, the sample size was for valid and complete cases.

**Lines 144–146:** in addition to the link to the DHS methodology good to **cite one or two standard DHS methodology references** (e.g., Rutstein and Rojas, DHS Comparative Reports).

**Response:** In the DHS website, everything is there. You can refer for more detailed matters.

**Lines 149–157 and 186–199**: ECIAF is described in multiple places, with some **redundancy**. These parts should be **merged and streamlined**.

**Response:** We have tried to revise but we have kept the important once.

**Line 150**: Typo—“W/H>22 Z-score” should likely be “W/H > +2 Z-score” (or BMI/A > +2 SD).

**Response:** Thank you very much. We have edited it.

**Line 152**: The sentence "subtracting children without anthropometric failure...from the sum..." is confusing. Mathematically, this explanation doesn’t make sense. What is meant is likely that **ECIAF = 1 if a child falls into any failure group (B to H), otherwise 0.**

**Response:** We have corrected it.

Check font type consistency: line 161 to 168

**Response:** We have edited. Please look at on the final manuscript.

* General: Add the survey years for each country or the range of years to understand temporal comparability.

**Response:** The survey years are included for each country. Please refer to the supplementary file (S1).

*Consider including how missing data were handled and whether any adjustments were made to account for survey design effects (e.g., use of Stata svy commands or multilevel modeling).

**Response:** To ensure representativeness and valid statistical inference, **survey weights, strata, and primary sampling units (PSUs)** were applied using **Stata's svyset command**. For descriptive statistics, estimates were computed using the **svy prefix** to account for the **complex survey design**.

For inferential analysis, a **multilevel binary logistic regression model** was fitted, accounting for clustering at the enumeration area (EA) level to adjust for intra-cluster correlations. Model comparisons were conducted using deviance (−2LL), Akaike Information Criterion (AIC), and Bayesian Information Criterion (BIC). The final model included both **individual- and community-level predictors**, with results reported as adjusted odds ratios (AORs) and 95% confidence intervals. Collinearity diagnostics (VIF < 10) confirmed no multicollinearity among predictors.

*Mention data use permission, to demonstrate compliance with data governance standards.

**Response:** An authorization letter was received after submitting a research description plan.

**Lines 190–198**: Several category labels are inconsistently capitalized (e.g., underweight and wasting vs. wasting and underweight), and the **sequence of labels (F, Y, G, H)** is not logically arranged. Also, “F: stunting only” is described differently in the narrative than in the table, **ensure consistency**.

**Response:** Sorry for the confusion that causes you. We have corrected.

**Lines 200–202**: The table is useful, but formatting needs improvement for clarity (e.g., align headers, avoid text breaking mid-sentence). Add a **proper caption** and clarify all acronyms (e.g., DE likely means SD).

**Response:** We have edited.

ECIAF calculation line (**line 200**) is imprecise. It should be:

ECIAF = % of children falling into any of the categories B through H.

**Response:** Corrected.

**Terminology**: Avoid inconsistent phrasing like “community women education” (line 167); revise to “community-level women’s education.”

**Response:** Corrected.

**Lines 160–172**: Some variables (e.g., media exposure, wealth index) are repeated or categorized inconsistently across lines.

**Response:** We have edited.

**Lines 174–177**: Appears to be a continuation of individual-level variables but is disconnected from prior sentences**,** integrate **and structure this list more clearly.**

**Response:** We have corrected.

**Line 203–209**: Good to define Africa regions, but this information might be better placed in the Methods or Supplementary Information, not under “Measurement variables.”

**Response:** We have revised it. Please the supplementary file (S1).

**Lines 210–212** (Community poverty) and 213–215 (Community literacy): the method of aggregating and dichotomizing the variables (e.g., "median split") may oversimplify continuous variation and should be justified in analysis or discussion.

**Response:** It is clear. Just it is not to make bulky.

**Line 217**: "Cleaned, appended, recoded" is vague. Specify **what variables were recoded, how missing data were handled, and criteria for data cleaning or exclusion.**

**Response:** It is clear. Just it is not to make bulky. You can see other published research articles.

**Line 219**: The sentence is poorly structured—"before the analysis the by using" needs correction. Suggested rewrite:

"Data were weighted prior to analysis using sampling weights, primary sampling units, and stratification variables to ensure national representativeness."

**Response:** Thank you very much for your suggestions. We have corrected it.

**Lines 232–234** repeat what was already stated earlier (lines 219–220). This redundancy should be eliminated or merged into one cohesive paragraph.

**Response:** We have eliminated the redundancy.

**Line 233**: Make clear **how weighting was implemented** in the multilevel regression (e.g., using svy commands, or incorporated into mixed models).

**Response:** For the multilevel binary logistic regression analysis, **survey weights were incorporated using the pweight option within the melogit command in Stata**, following DHS guidelines for multilevel modeling. Clustering at the enumeration area (EA) level was modeled as a random intercept to account for hierarchical data structure.

Lines 221–224: Clarify the levels of the model (e.g., children nested within clusters or countries?).

**Response: T**o account for the hierarchical nature of the DHS data, a **two-level mixed-effects logistic regression model** was applied. In this model, **children were nested within clusters or enumeration areas (EAs)**, which represent the primary sampling units (PSUs) in the DHS design. Random intercepts were included at the cluster level to account for unobserved heterogeneity and intra-cluster correlation.

*****Specify whether random intercept or random slope models were used.

**Response:** Both random intercept and random slope models were used.

**Line 224**: “Bivariable” should be corrected to **"bivariate”**

**Response:** We have edited.

**Lines 225–227**: The variable selection process (p < 0.2, literature, and clinical expertise) is reasonable, but **"clinical expertise"** is ambiguous in secondary data analysis. Clarify whether domain experts were consulted or if prior empirical findings guided this.

**Response:** We have revised the manuscript. Please see the final version of the manuscript.

Lines 228–243: Some definitions are copied twice (see lines 228–230 and 236–243), which is redundant.

**Response:** We have avoided the redundancy.

*****Formula for ICC (line 240) is correct but should be introduced with clarity.

**Response:** W have stated it.

*Define PCV (Proportional Change in Variance)—it's mentioned in line 229 but never explained.

**Response:** Now we have explained the PCV very well.

**Line 239**: "WRA" (women of reproductive age) seems out of context here. This study focuses on children under five, so this may be an **editing error.**

**Response:** Corrected. It was an editorial error.

Lines 244–249: Clarify whether random effects were included in all models, and how the final model controlled for country- or cluster-level heterogeneity.

**Response:** Random effects were included in **all four models** to account for the hierarchical structure of the data, with **children nested within clusters (enumeration areas).** While regional variables were used to partially control for **between-country variation,** country itself was not modeled as a separate random effect due to data structure limitations.

*The use of log-likelihood ratio and deviance is appropriate. You may consider also reporting AIC/BIC for model comparison.

**Response:** We have already documented in the table form. Please have a look at on the manuscript again.

**Line 253**: “clustrs” is a typo; should be “clusters

**Response:** Corrected.

Lines 255–260: You may **explicitly mention** that permission to use DHS data was obtained via registration on [DHS program website](https://dhsprogram.com).

**Response:** For an evidence, the authorization letter is attached as supplementary file (S2)

**Comment #5.**

Result

Lines 262–270: Study population characteristics: Line 268: Change "Northern African countries" to "Northern African country" to match singular form (only Mauritania).

**Response:** Dear Reviewer, thank you very much for your genuine feedbacks. We have corrected it.

Line 273: “After recoded of the child age majorities” is grammatically awkward. Consider: “After recoding child age, the majority (57.09%) were 24–59 months…”

**Response:** Thank you very much for your insightful feedbacks. We have revised it.

Lines 271–278: Basic descriptive statistics: Inconsistent use of past and present tense throughout. Use **past tense** consistently for reporting survey results.

**Response:** Updated.

*Consider specifying “weighted percentage” for clarity when presenting weighted data.

**Response:** All are weighted values.

Lines 279–278: Table 2: Line 274: “nearly three-fourth (64.09%)” → should be “nearly two-thirds

**Response:** Corrected.

Line 275: Typo: “64.09% of were in children…” → remove “of” or rewrite for clarity.

**Response:** We have made correction.

Lines 281–288: Prevalence of ECIAF by characteristics: Consider using Pearson’s Chi-square p-values in Table 3 to denote statistically significant differences in ECIAF prevalence by characteristics.

**Response:** We believe that putting Pearson’s Chi-square p-values in Table 3 in unnecessary because we have performed regression analysis in advance.

* Use consistent formatting for n (%) across all entries.

**Response:** We have done it.

Lines 290–300: Pooled prevalence: Line 293: “Burundi 59%… and Gabon 20%…” → Reword to: “Prevalence ranged from 59% in Burundi (95% CI: 58–60%) to 20% in Gabon (95% CI: 19–21%).”

**Response:** Updated.

* “Figure 1” should be referenced properly with an in-text caption and axis explanation.

**Response:** Corrected.

Lines 301–319: Subgroup analysis by region: Line 310: “Sothen Africa” should be corrected to “Southern Africa”.

**Response:** We have corrected it.

* Sentence structures in Lines 304–314 are repetitive and sometimes unclear. Suggested revision: Present findings in bullet points or tabular format to avoid long sentences.

**Response:** We have revised it. Please have a look at on it.

* Line 309: “lowest both countries…” is grammatically incorrect. Consider: “The lowest prevalence was observed in Ghana and Gambia (both 24%).”

**Response:** We have revised it.

Lines 320–368: Fixed effect analysis:

*Use consistent terminology: “higher odds of ECIAF” or “increased risk” do not alternate between both in the same paragraph.

**Response:** We have checked it.

*Clarify the reference categories in-text where not obvious (e.g., for household size, parity, education).

**Response:** We have corrected it.

* Line 342: “has comorbidity” → revise to “had comorbidity”.

**Response:** Corrected as per the feedback.

*Line 348: Minor grammatical issue: “has no antenatal care visit” → revise to “did not have ANC visits”.

**Response:** Corrected as “did not have ANC visits”.

*Line 357: “3–5 under-5 children” is confusing—does it mean 3 to 5 under-5s or 3 to 5 children total? Clarify.

**Response:** It is to mean 3 to 5 under-5s.

**Lines 369–372: Table 4**: Table 4 needs to be formatted carefully and consistently across models. Some AORs have inconsistent spacing or punctuation.

**Response:** The manuscript is revised.

* Table title (Line 370) should specify that results are from a multilevel logistic regression.

**Response:** We have revised the manuscript.

Lines 374–389: Random effect analysis and model fit statistics: Line 381: Typo in parenthesis – “i.e one from a cluster…” → “i.e., one from a cluster…”

**Response:** Thank you very much for your certain observation. We have corrected it.

Line 385: “63.58% of the variability in the ECIAF…” → “of the variability in ECIAF” (drop “the”).

**Response:** Thank you very much for your certain observation. We have corrected it.

Lines 390–392: Table 5: Table 5 title says “in Ethiopia” (Line 392), but this is a multi-country study. Correct to “in Sub-Saharan Africa”.

**Response:** Great observation. We have corrected it.

**Comment #6.**

Discussion

Lines 395–400: Introduction of findings: Line 400: Awkward phrasing: “To compare the finding…there was no similar research…” → Recommend:
“There is limited prior research using ECIAF in the same context; however, our findings align with CIAF-based studies.”

**Response:** Thank you very much for your constructive feedbacks. We have made corrections.

Lines 401–405: Comparison with other studies: But, this prevalence aligns…” → Remove “But,” for better flow.

**Response:** We removed it.

Line 404: Avoid starting a sentence with “While” when not showing contrast.
Suggest: “A study in Bangladesh reported a CIAF prevalence of 48.3%...”

**Response:** We have revised.

Lines 406–415: Regional disparities: Line 411: “ranging from 44% in Angola to 38% in South Africa” – the wording is fine, but the contrast is weak. Consider: *“...with Angola (44%) reporting the highest, and South Africa (38%) the lowest within the region.”*

**Response:** We have updated.

* Line 415: “Male children were more affected…” → Good point, but link to mechanism or literature earlier.

**Response:** We have linked to mechanism and earlier literature.

Lines 416–424: Multilevel findings overview: Line 424: End punctuation is a misplaced superscript “33.” Should either be removed or properly cited.

**Response:** We have edited.

Lines 425–437: Detailed associations: **Split this into subheadings or separate paragraphs** (e.g., child-level, maternal, household, community factors).

**Response:** We believe it is correct description.

* Line 426: “child birth average size, small size…” → redundant. Suggest condensing.

**Response:** Both child birth average size and small size are different categories.

* Line 430: “and negatively were institutional delivery…” → revise: “...and were negatively associated with ECIAF: institutional delivery, ANC visits, and grand multiparity.”

**Response:** We accepted the feedback and corrected it.

* Avoid repeating “positively associated with the risk…” in every clause. Group ideas logically.

**Response:** We have edited.

Lines 438–452: Interpretations: *Line 446: “short birth intervals (<24 months) also had greater risk” → “also carried greater risk”

**Response:** Corrected

*Line 448–450: “Perceived birth size…” and “corroborating evidence…” – Good use of literature.

**Response:** Already cited.

Lines 453–460: Maternal characteristics: Line 459: Consider clarifying how “grand multiparity” being protective differs from what’s found in other literature.

**Response:** We have explained the possible reasons.

Lines 461–467: Household characteristics: Line 466: “Conversely, larger family sizes…” – Provide potential mechanism briefly.

* Consider distinguishing between family size and number of under-5s for clarity.

**Response:** It is obvious they are different issues.

Lines 468–477: Community-level and regional factors: Line 475: “Southern, Western, Eastern, and Central Africa…” → Consider reordering by prevalence or alphabetically.

**Response:** Corrected by Alphabetical order.

Lines 478–483: Implications: Slight repetition with applications section. Consider integrating or reducing overlap.

**Response:** We have updated.

Lines 484–496: Applications: Line 486: Start with stronger topic sentence: “The findings have several practical implications for child health policy and programming.”

**Response:** WE have accepted the feedback.

* Line 494: “wealth redistribution strategies” – might be too politically charged for some journals; consider: “economic empowerment initiatives”.

**Response:** We have revised it.

Lines 497–503: Health system: Avoid overly long sentences (e.g., Line 499–500).

*Consider mentioning health workforce training or integration of nutrition into routine services.

Lines 504–509: Community-based programs: Could benefit from examples or references from successful case studies.

Lines 510–516: Research: Line 513: “Longitudinal studies…” – clarify why they are particularly important (e.g., to establish causality).

Line 517: Wrap-up Sentence: Incomplete sentence due to cutoff. Consider completing as:
“...and guide future programming to reduce child malnutrition in Sub-Saharan Africa.”

**Response:** This section is updated entirely.

**We would like to thank the reviewers and editors for evaluating our manuscript. We have tried to address all the concerns in a proper way and believe that our paper has been improved considerably. We would be happy to make further corrections if necessary and look forward to hearing from you all soon.**

I hope that the revised manuscript is accepted for publication in PLoS ONE.

Sincerely yours,

Amare Muche (Corresponding author)

[Amaremu7@gmail.com](mailto:Amaremu7@gmail.com)
