# Supplementary material for: Unveiling the full picture of malnutrition in Sub-Saharan Africa: The extended composite index of anthropometric failure among children under-5 in the SDG era
Source: PLoS One. 2025 Oct 3;20(10):e0333785. doi: 10.1371/journal.pone.0333785 (PMC12494244; doi:10.1371/journal.pone.0333785)
Supplement: Attachment — Submitted filename: Response_to_reviewers_auresp_2.docx [file pone.0333785.s006.docx]

**Date: September 08, 2025**

**Rebuttal letter**

**Response to the editor questions**

**Question #1.**

Line 23-33: The "objective of the study" statement is either missing or not clearly stated

**Response:** Thank you for this remark. We have stated the objective of the study (**Please see the revised version of the manuscript**).

**Question #2.**

Line 34-35: I would like you to revise the sentence to show that you conducted a secondary data analysis research instead of saying that you conducted a community-based cross-sectional survey, since you did not collect the primary data.

**Response:** Thanks again for your positive feedback. We made a correction of the manuscript. (**Please look at the updated version of the manuscript**).

**Question #3.**

Line 55: There is a typographical error: "children-5", it should be "children under-5"

**Response:** We have Corrected it. **Please have a look at the revise version of the manuscript.**

**Question #4.**

Line 44-47: I suggest you include all the key factors associated with increased odds for ECIAF: **Increasing child age (6-23 months [AOR: 1.56, p<0.001] & 24-59 months [2.03, p<0.001]), multiple birth [2.38, p<0.001], reducing birth size (Average [AOR: 1.20, p<0.001] & Small [1.80, p<0.001]), having comorbidity [AOR: 1.12, p<0.001], reducing level of mother's educational status (Primary [AOR: 1.20, p<0.001] & No formal [1.36, p<0.001]), increasing number of children under-5 in household (2 children [AOR: 1.10, p<0.001] & 3-5 children [1.14, p<0.001]), reducing household wealth status (Rich [AOR: 1.28, p<0.001], Middle [1.31, p<0.001], Poor [1.42, p<0.001], & Poorest [1.45, p<0.001]), living in rural area [AOR:1.15, p<0.001], and having no media exposure [1.15, p<0.001]**.

**Response:** We have corrected the manuscript. **Please have a look at the revise version of the manuscript.**

**Question #5.**

Line 51-55: The statement "Child sex, child age, type of birth, preceding birth interval, perceived sizes at birth, comorbidity, educational level of the mother, parity, place of delivery, antenatal care visit, wealth index, family size, number of under-5 children, place of residence, media exposure and Africa region were identified factors associated with ECIAF." is totally out of place, and irrelevant. It should be expunged.

**Response:** We have deleted the recommended section.

**Question #6.**

Line 55-59: **These statements should be revised to incorporate a set of Nutrition sensitive interventions that will primarily address the risk factors mentioned in the result section and inadvertently reduce the prevalence of ECIAF in the long run**. Perhaps these articles may help:

<https://doi.org/10.1016/> S2214-109X(23)00562-4

<https://doi.org/10.1186/s40795-021-00443-1>

**Response:** Now, the paper is updated.

**Question #7.**

Line 134: Kindly include the "objective of the study" statement.

**Response:** We have included the objective of the study in the recommended section of the manuscript.

**Question #8.**

Line 221-223: To avoid ambiguity and speculation among the readers, I advice you revise these lines to clearly show that you are referring to the primary surveys (DHS) that produced the dataset you used for this secondary data analysis research.  For instance, Line 222-223: **"The surveys utilized a two-stage stratified cluster sampling technique to ensure nationally representative estimates across 26 Sub-Saharan African (SSA) countries."**

**Response:** We have revised the manuscript. **Please have a look at the revise version of the manuscript.**

**Question #9.**

Line 375-377: The statement in these lines needs to be reviewed for correctness. "The odds of having ECIAF was higher among children under-5 having perceived average birth size (AOR: 1.20; 95% CI: 1.14, 1.26) and perceived small birth size (AOR: 1.80; 95% CI: 1.68, 1.93) as ***compared with those whose ages were below 6 months.***"

**Response:** We have reviewed and corrected it.

**Question #10.**

Line 366 & 380: Maintain consistency: "The odds of having ECIAF" instead of "The risk of having ECIAF"

**Response:** We have corrected it.

**Question #11.**

Line 383-387: Please revise the last segment of the statement to read as follows: ".....**as compared to primiparity were however significantly protective against the odds of having ECIAF in children under-5 years in SSA**".

**Response:** We have revised it.

**Question #12.**

Line 388: You might need to include "**Concerning household level factors**," in the opening statement of the paragraph.

**Response:** We have updated it.

**Question #13.**

Line 388-399: The statement needs revision as follows: **"Children under-5 from rich households(AOR: 1.28; 95% CI: 1.17, 1.41), middle households(AOR: 1.31; 95% CI: 1.19, 1.44), poor households(AOR: 1.42; 95% CI: 1.28, 1.56)  and poorest households (AOR: 1.45; 95% CI: 1.31, 1.61), respectively had higher odds of ECIAF as compared to those from richest households"**.

**Response:** we have revised based on the recommendation.

**Question #14.**

Line 391-396: The statement requires revision: **Other household factors such as having 2 (AOR: 1.17; 95% CI: 1.10, 1.23 ) or 3-5  (AOR: 1.14; 95% CI: 1.06, 1.22) children under-5 in the household as compared to 1 under-5 child was associated with odds of having ECIAF; while having 5-9 member (AOR: 0.92; 95% CI: 0.87, 0.98) or greater than 9 family members (AOR: 0.90; 95% CI: 0.83, 0.98) as compared to 1-4 family members, was protective against the odds of having ECIAF**.

**Response:** The manuscript is revised right now.

**Question #15.**

Line 397-404: Statements requires revision: **"With regards to the community level factors, children under-5 from Southern African countries (AOR: 2.01; 95% CI: 1.80, 2.24), Western African countries (AOR: 1.37; 95% CI: 1.24, 1.51), Eastern African countries (AOR: 1.44; 95% CI: 1.30, 1.60), and Central African countries (AOR: 1.97; 95% CI: 1.75, 2.22), respectively had significantly higher odds of ECIAF than the Northern African countries. Other factors such as living in rural residence (AOR: 1.15; 95% CI: 1.09, 1.22) versus urban residence, and exposure to media compared no exposure to media (AOR: 1.15; 95% CI: 1.10, 1.20) exposure were significantly associated with higher odds of ECIAF in children under-5 years old in SSA"**.

**Response:** We have revised the manuscript according to your recommendations.

**Question #16.**

Lastly in the discussion section, I would like you to carefully consider realigning or combining the statements in the following lines to ensure consistency and flow before delving into discussing how "Male children were more affected than females":

Line 451-452: "The study also highlights several factors associated with higher ECIAF prevalence."

**Response:** We have updated the manuscript.

**Question #17.**

Line 459-467: "The current study also examined factors associated with the Extended Composite Index of Anthropometric Failure (ECIAF) among children under five years old in sub-Saharan Africa (SSA) using a multilevel modeling approach. The findings highlight the significant roles of child, maternal, household, and community-level factors in determining ECIAF. In our study child age, child sex, child size at birth, child birth type, child birth order, child birth interval, child having comorbidity, mother’s education, parity, antenatal care visit, place of delivery, household wealth index, family size, number of under-5 children in the household, African region, place of residence and media exposure were significantly associated with ECIAF in children under-5 years old in SSA."

**Response:** We thank you for the constructive and genuine feedbacks. We have revised manuscript. **Please have a look at the revise version of the manuscript.**

**Response to the journal requirements questions**

**Question #1.**

If the reviewer comments include a recommendation to cite specific previously published works, please review and evaluate these publications to determine whether they are relevant and should be cited. There is no requirement to cite these works unless the editor has indicated otherwise.

**Response:** We have done our revisions based on the comments provided.

**Question #2.**

Please review your reference list to ensure that it is complete and correct. If you have cited papers that have been retracted, please include the rationale for doing so in the manuscript text, or remove these references and replace them with relevant current references. Any changes to the reference list should be mentioned in the rebuttal letter that accompanies your revised manuscript. If you need to cite a retracted article, indicate the article’s retracted status in the References list and also include a citation and full reference for the retraction notice.

**Response:** We have seen the references. There are no retracted papers cited there.

**Question #3.**

While revising your submission, please upload your figure files to the Preflight Analysis and Conversion Engine (PACE) digital diagnostic tool, [https://pacev2.apexcovantage.com/](https://track.editorialmanager.com/CL0/https:%2F%2Fpacev2.apexcovantage.com%2F/1/010f019926fb71f7-66aeab07-1ae2-4a0e-a6db-87ba705d8aa3-000000/nQhN1OcUkfIHQqQ2if9XU4pv8WMD_cHhwZqebnIBkJU=227). PACE helps ensure that figures meet PLOS requirements. To use PACE, you must first register as a user. Registration is free. Then, login and navigate to the UPLOAD tab, where you will find detailed instructions on how to use the tool. If you encounter any issues or have any questions when using PACE, please email PLOS at [figures@plos.org](mailto:figures@plos.org). Please note that Supporting Information files do not need this step.

**Response:** We have done it.

**We would like to thank the editors and reviewers for evaluating our manuscript. We have tried to address all the concerns in a proper way and believe that our paper has been improved considerably. We would be happy to make further corrections if necessary and look forward to hearing from you all soon.**

I hope that the revised manuscript is accepted for publication in PLoS ONE.

Sincerely yours,

Amare Muche (Corresponding author)

[Amaremu7@gmail.com](mailto:Amaremu7@gmail.com)
